# Effects of a 90-min educational intervention for patients with insect venom allergy: a prospective controlled pilot study

**DOI:** 10.1186/s13223-021-00524-7

**Published:** 2021-02-25

**Authors:** Lisa-Sophie Schoeben, Nicole Mohr, Corinna Bubak, Astrid Schmieder, Marthe-Lisa Schaarschmidt

**Affiliations:** 1 Department of Dermatology, University Medical Center Mannheim, Heidelberg University, Theodor-Kutzer-Ufer 1-3, 68135 Mannheim, Germany; 2 Institute for Health Services Research in Dermatology and Nursing (IVDP), University Medical Center Hamburg-Eppendorf, Hamburg, Germany

**Keywords:** Anaphylaxis, Emergency medication, Insect venom allergy, Patient education, Emergency management

## Abstract

**Background:**

Anaphylactic sting reactions need a prompt management. A structured educational intervention for patients with insect sting allergy has not been implemented so far. The purpose of this study was to analyze the effects of a structured 90-min educational intervention for patients with insect sting allergy.

**Methods:**

Patients with an insect venom allergy were offered to participate in a structured 90-min group education (intervention group (IG)) or to attend a control group (CG). The patients’ subjective self-assurance in using the emergency medication, the willingness to always carry the emergency medication, the mental health status, absolute one-time willingness-to-pay (WTP) for complete cure, a disease knowledge assessment and a simulation test to examine the ability to manage an acute sting reaction were estimated at baseline (t0) and at follow-up (t1) as outcome parameters.

**Results:**

55 patients participated in the IG (n = 25, 52.0% female, mean age 55.9 years) or the CG (n = 30, 56.7% female, mean age 52.0 years). Both arms showed a significant gain in self-assurance in using the emergency medication (IG: 6.1 at t0 vs. 8.6 at t1, p < 0.0001 and CG: 7.1 vs. 8.0, p = 0.0062) and ability to manage an acute sting reaction (IG: 6.7 vs. 11.4, p < 0.0001 and CG: 9.0 vs. 10.5, p = 0.0002) at t1. However, trained participants showed a significantly higher gain in the respective parameters. There were no significant changes regarding the remaining examined outcome parameters.

**Conclusions:**

Patients who are willing to invest 90 min in a patient education intervention benefit significantly by an increased subjective and objective empowerment to manage an acute sting reaction.

## Background

Anaphylaxis is a clinical emergency of rapid onset with symptoms of an immediate-type allergic reaction [1]. Clinical features range from mere skin involvement to mild, moderate or severe, life-threatening systemic symptoms according to the German S2-guideline for acute therapy and management of anaphylaxis [1]. However, allergic reactions are not only physically frightening but may also cause a psychological burden [2]. In adults, one of the most common causes of anaphylaxis are insect stings [3, 4]. Even though fatal sting reactions are infrequently reported, several sting fatalities may be unrecognised and attributed to other causes [5]. In untreated adults with a history of systemic sting reactions, a repeated sting causes another systemic reaction in about 30–65% of cases [6]. Therefore, all patients with a history of a previous systemic allergic reaction triggered by a stinging insect should receive information about how to avoid a re-sting and how to recognize anaphylaxis. Furthermore, the indication for venom immunotherapy (VIT) needs to be assessed by a specialist [7].

Acute sting reactions need prompt management. Thus, all patients who survived an anaphylaxis episode and cannot avoid with certainty the elicitor should be prescribed an “emergency set for self-help “(in Germany, Austria and Switzerland commonly the following drugs are included: epinephrine auto-injectors (EAI), oral H1-antihistamines and oral glucocorticoids) and should be advised to carry it with them [1]. Moreover, an adequate instruction about how to correctly use the kit for self-treatment is of particular importance and patients must understand that using the kit is not a substitute for emergency medical attention (in case of biphasic or protracted anaphylaxis) [1, 4]. Summing up, given the serious consequences for untreated anaphylactic reactions or the misuse of the emergency medication, adequate patient education regarding anaphylaxis is crucial. However, poor knowledge on how to use the emergency kit and poor compliance in carrying the emergency medication have been frequently reported [8–10] and the need for better patient education is repeatedly highlighted [7–16].

Interestingly, Brockow et al. investigated the effects of a structured patient education intervention on patients with previous episodes of anaphylaxis and caregivers of affected children [17]. The two 3-h schooling modules of group education had a significant impact on patient knowledge, increased their emergency management competence and reduced caregiver anxiety. The most common trigger for anaphylaxis was food in their study cohort. Based on those findings, we suggested that a shorter patient education specifically addressing patients with insect sting allergy may be beneficial, too.

With the presented prospective pilot study, we evaluated whether a 90-min group-based educational program especially for patients with insect sting allergy also has a positive effect.

## Methods

### Patients

The study was conducted at the Department of Dermatology, Venereology and Allergology at the University Medical Center Mannheim, Germany from 04/2016 to 01/2017. The division for Allergology offers diagnostic workup and VIT for patients with insect sting allergy. Every patient with a history of an anaphylactic sting reaction receives non-standardized short oral and written instructions on how to avoid stings and how to use the emergency medication as well as a practical demonstration of the EAI with a training device once at the first visit. The explanation and demonstration take approximately 5 min.

All patients visiting the department because of an anaphylactic insect sting reaction were asked to participate in the study. Inclusion criteria comprised age ≥ 18 years and diagnosis of an anaphylactic reaction after an insect sting confirmed by an allergist (defined as a history of insect venom-related anaphylaxis and evidence of immunoglobulin E (IgE)-mediated sensitization to the insect). The routine diagnostic workup included skin tests (skin prick testing (venom concentrations of 1.0 μg/mL, 10 μg/mL and 100 μg/mL) and if skin prick testing is negative intradermal testing (venom concentrations of 0.01 μg/mL, 0.1 μg/mL, and 1.0 μg/mL)) and measurement of specific IgE. Only if the aforementioned tests were all negative but there was an unequivocal history of insect venom-related anaphylaxis the basophil activation test was used. A sensitization was considered verified, if skin test and/or laboratory tests were positive. An exclusion criterion was inability to consent. After written informed consent, the participants were allowed to choose between the intervention group (IG) receiving a 90-min educational intervention and the control group (CG). The study was performed in accordance with the latest revision of the Helsinki Declaration and received approval by the Ethics Committee of the Medical Faculty Mannheim (reference no. 2016-580 N-MA).

### Data collection

Participants of the IG were asked to complete a paper-based questionnaire and a short simulation test after study inclusion 2–4 weeks before the educational intervention (baseline = t0) and 8–12 weeks after the educational intervention at a regular visit (follow-up = t1). The CG completed the same questionnaires and simulation test as the IG also directly after study inclusion (t0) and about 8–12 weeks later during a regular visit (t1).

To measure the beneficial effect of the intervention, the questionnaires comprised several outcome parameters of interest. Firstly, it contained visual analogue scales (VAS) regarding the patients’ subjective self-assurance in using the emergency medication (range: 0 (very unsure) to 10 (very sure)) and the patients’ willingness to always carry the emergency medication (range: 0 (not at all willing) to 10 (very willing)). Secondly, the individual depression and anxiety levels were assessed with the Hospital Anxiety and Depression Scale (HADS) [18]. This scale is a validated and a commonly used tool to assess patients’ mental status and performs well in screening for anxiety or depression disorders [19]. Thirdly, participants were asked for the absolute one-time willingness-to-pay (WTP) for a complete cure of the insect venom allergy to measure the disease burden. Fourthly, a short quiz including questions on the emergency medication set (3 items), on medication that should be avoided during the VIT (1 item), and regarding further steps after administration of the emergency medication (1 item) (maximum total score 12 points) was also part of the questionnaires to quantify the individual state of knowledge (for details see Additional file 1: Table S1).

In addition to the questionnaire, a short simulation test was used to examine patients’ ability to manage an acute sting reaction at t0 and t1. Therefore, the participant was confronted with a theoretical anaphylactic reaction after an insect sting and asked to tell in depth what to do step by step including demonstration of auto-injector use with an EAI training device. The performance was rated in a standardized fashion by AS, LS and MS together according to a predetermined point-based system with a maximum total score of 13 points (for details see Additional file 2: Table S2). Moreover, at baseline, the survey included questions on demographics and medical history and the physician documented the venom involved as well as the severity of reaction according to the Mueller 4-grade classification [20].

### Educational intervention

The IG received a 90-min patient education intervention dealing with knowledge about anaphylaxis (trigger, pathogenesis, clinical features, course), information about insect stings (prevalence, differentiation of bees and wasps, sting prevention), and sting reaction management (components of the emergency kit, how to correctly use the kit for self-treatment, need for emergency medical attention, emergency action plan). Finally, the administration of the EIA was demonstrated and practiced in detail with the participants using training devices. Subsequently and during the intervention, participants were encouraged to ask questions. The training was held by two senior residents of the Department of Dermatology, Venereology and Allergology at the University Medical Center Mannheim (PD Dr. ML. Schaarschmidt and Prof. Dr. A. Schmieder), both experienced in the field for years. The intervention was offered 3 times between August and October 2016 with a median of n = 8 (range: 7–10) participants.

### Statistical analyses

GraphPad Software (GraphPad Software, Inc., La Jolla, CA 92,037 USA) and IBM SPSS Statistics (Version 25 for Windows, IBM Germany, Ehningen, Germany) were used for analyses. If Gaussian distribution was confirmed with the d’Agostino and Pearson omnibus normality test, statistically significant differences for continuous parameters were examined by unpaired t-tests. For all continuous parameters without Gaussian distribution, the Mann–Whitney test was performed. For Categorial variables the Chi Square test was used as indicated. Analyses of covariance were conducted to compare differences in the outcome variables (t1—t0) between the 2 arms (covariate = baseline values of the respective variable). Data were analyzed by using intention-to-treat analysis. Missing values at t1 were estimated by using “last observation carried forward” (LOCF). Significance was assumed at p < 0.05. Effect size > 0.1 suggests a weak effect, > 0.25 a medium effect, and > 0.4 a strong effect.

## Results

### Participant characteristics

In total, 57 patients gave written informed consent. One patient had to be excluded due to a largely incomplete questionnaire and a second patient because there was no evidence of IgE-mediated sensitization to the venom of the culprit insect (skin test, specific IgE test as well as basophil activation test were negative). Thus, 55 participants (54.5% female) were included in the final analysis (regarding baseline characteristics of the whole study cohort see also Schaarschmidt et al. [21] and Schoeben et al. [22]). 25 patients were part of the IG (52.0% females) and 30 part of the CG (56.7% females; Table 1). The mean age (IG: 55.9 years, CG: 52.0 years) and the mean disease duration (IG: 5.0 years, CG: 5.3 years) were comparable between the groups. Furthermore, the severity of anaphylaxis did not differ significantly between the arms. The main allergen reported was wasp venom (IG: 84.0%, CG: 80.0%) and most participants were currently on VIT (IG: 72.0%, CG: 90.0%). The duration of VIT did not differ significantly between the groups (IG: 33.6 months, CG: 23.5 months).

**Table 1:** Baseline characteristics of the study cohort

	Total	Intervention Group	Control Group	p
	n = 55	n = 25	n = 30	
Females, n (%)	30 (54.5)	13 (52.0)	17 (56.7)	0.739^a^
Age (years)				
Mean ± SD Min–max	53.8 ± 12.1 23–77	55.9 ± 13.4 23–77	52 ± 10.8 35–75	0.236^b^
Disease duration (years)				
Mean ± SD Min–max	5.1 ± 6.1 1–30	5 ± 3.8 1–15	5.3 ± 7.6 1–30	0.326^a^
Venom involved, n (%)				
Honeybee Wasp Honeybee and wasp	7 (12.7) 45 (81.8) 3 (5.5)	2 (8.0) 21 (84.0) 2 (8.0)	5 (16.7) 24 (80.0) 1 (3.3)	0.503^c^
Severity of reaction, n (%)				
Mueller grade I Mueller grade II Mueller grade III Mueller grade IV	6 (10.9) 25 (45.5) 20 (36.4) 4 (7.3)	2 (8.0) 11 (44.0) 9 (36.0) 3 (12.0)	4 (13.3) 14 (46.7) 11 (36.7) 1 (3.3)	0.618^c^
Venom immune therapy status, n (%)				
Currently ongoing Completed Intended Not intended	45 (81.8) 2 (3.6) 3 (5.5) 5 (9.1)	18 (72.0) 1 (4.0) 3 (12.0) 3 (12.0)	27 (90.0) 1 (3.3) 0 (0) 2 (6.7)	0.205^c^
Venom immune therapy duration (months)				
Mean ± SD Min–max	27.6 ± 26.1 1–120	33.6 ± 33.6 1–120	23.5 ± 19.4 1–60	0.569^a^

Differences were tested for significance with ^a^ Mann–Whitney-U-Test, ^b^ unpaired t-test or ^c^ Chi-square test

Max: maximum; min: minimum; n: number; SD: standard deviation

### Pre-post comparison of outcome parameters

#### Subjective self-assurance in using the emergency medication

**Fig. 1:**
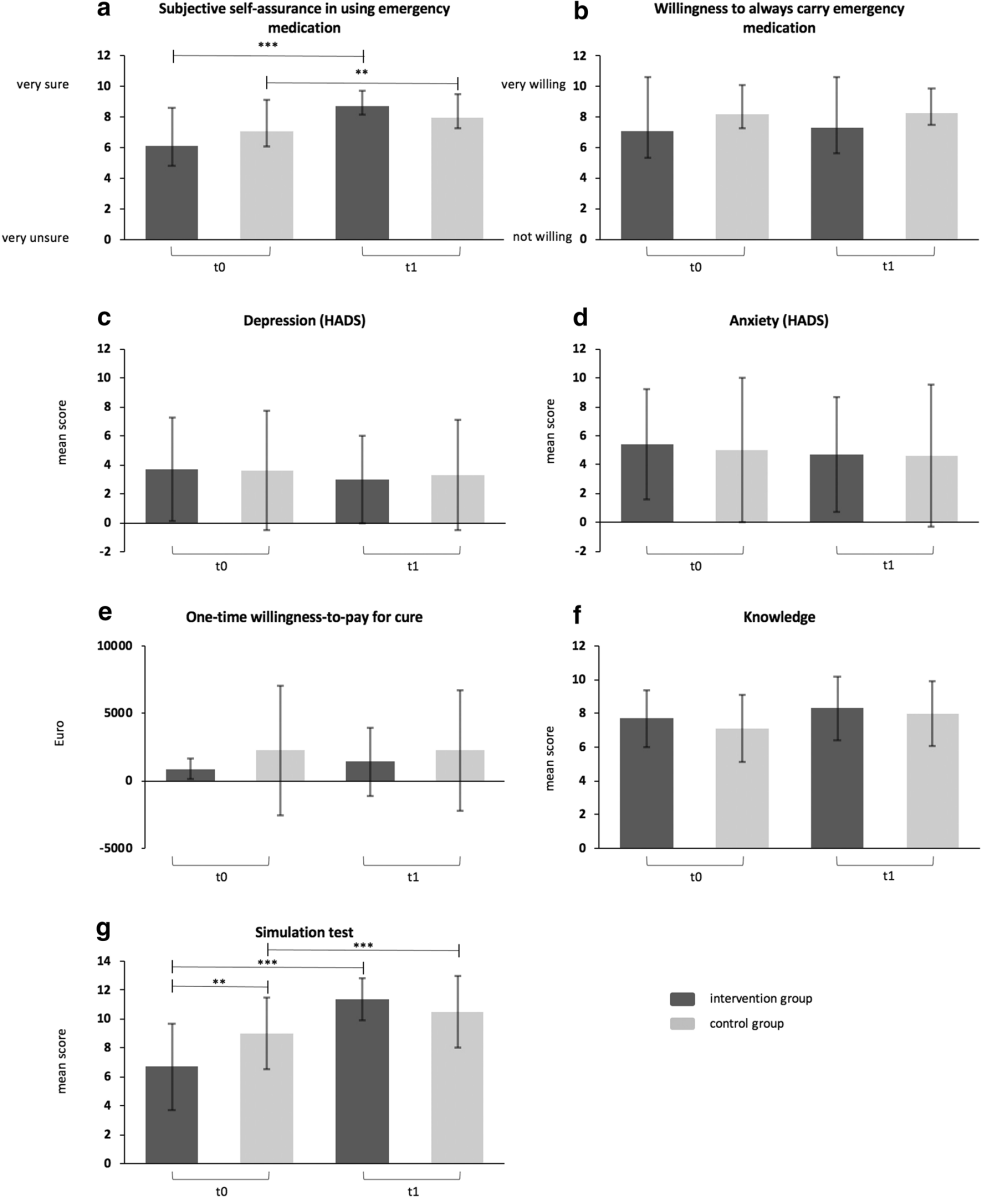
Pre (t0)—post (t1) comparison of the outcome parameters as well as comparison between the intervention and the control group. **a** Mean subjective self-assurance in using the emergency medication on a visual analogue scale (VAS) (range: 0 (very unsure) to 10 (very sure)). **b** Mean willingness to always carry the emergency medication on a VAS (range: 0 (not willing) to 10 (very willing)). **c** Mean depression level and **d** mean anxiety level regarding the Hospital Anxiety and Depression Scale (HADS). **e** Mean absolute one-time willingness to pay for complete cure (in Euro). **f** Mean score reached in the Knowledge-quiz (maximum score: 12 points). **g** Mean score achieved at the simulation test dealing with the patients’ ability to manage an acute sting reaction (maximum score: 13 points). **p ≤ 0.01, ***p ≤ 0.001. Bars: Means with standard deviation

At t0, mean values of subjective self-assurance did not differ significantly between the IG and the CG (IG (n = 25): 6.1 vs. CG (n = 30): 7.1, p = 0.109). At t1 subjective self-assurance had significantly increased for both arms (IG (n = 23): increase to 8.7 at t1, p < 0.0001; CG (n = 27): increase to 8.0 at t1, p = 0.006; Fig. 1a). However, the IG showed a statistically significantly higher gain in self-assurance than the CG at follow-up (differences t1 to t0 IG – difference t1 to t0 CG = 1.09 (95% confidence interval (95% CI) = 0.5; 1.7), p = 0.001; Table 2).

**Table 2:** Outcome variables by using Analyses of covariance (covariate = baseline values) of differences between baseline (t0) and follow-up (t1) for the Intervention Group and the Control Group

Outcome variables	Intervention Group (IG)	Control Group (CG)	IG- CG	p	Effect size (r)
	Estimated marginal means and 95% CI of differences (t1-t0)	Estimated marginal means and 95% CI of differences (t1-t0)	Estimated marginal means and 95% CI of differences ((IG t1-t0)—(CG t1-t0))		
	Mean (95% CI)	Mean (95% CI)	Mean (95% CI)		
Subjective self-assurance^a^	2.141 (1.71; 2.57)	1.049 (0.66; 1.44)	1.092 (0.50; 1.68)	*0.001*	0.457
Willingness to carry medication^b^	0.112 (− 0.54; 0.77)	0.373 (− 0.22; 0.97)	− 0.261 (− 1.19; 0.63)	0.560	0.081
Depression (HADS)	− 0.511 (− 1.20; 0.18)	− 0.008 (− 0.64; 0.63)	− 0.503 (− 1.44; 0.44)	0.288	0.147
Anxiety (HADS)	− 0.543 (− 1.45; 0.37)	− 0.114 (− 0.95; 0.72)	− 0.429 (− 1.66; 0.81)	0.488	0.096
One-time WTP^c^	41.055 (− 294; 376.1)	275.815 (6.64; 545.0)	− 234.76 (− 667.91; 198.4)	0.277	0.198
Knowledge quiz	0.555 (− 0.03; 1.14)	0.138 (− 0.40; 0.67)	0.417 (− 0.38; 1.21)	0.288	0.144
Simulation test	3.673 (2.86; 4.49)	2.106 (1.37; 2.84)	1.567 (0.43; 2.71)	*0.008*	0.357

CI: confidence interval. Effect size: > 0.1 weak effect, > 0.25 medium effect, > 0.4 strong effect. HADS: Hospital Anxiety and Depression Scale. €: Euro. t0: baseline. t1: follow-up. Significant findings are highlighted in italics

^a^Subjective self-assurance in using the emergency medication

^b^Willingness to always carry the emergency medication

^c^Absolute one-time willingness-to-pay (WTP) for a complete cure

#### Willingness to always carry the emergency medication

Mean willingness to always carry the emergency medication was comparable between the groups at baseline (IG (n = 25): 7.1 vs. CG (n = 30): 8.2, p = 0.149; Fig. 1b). Neither the IG nor the CG showed a significant higher willingness to always carry the emergency medication at follow-up (IG (n = 23): increase to 7.3 at t1, p = 0.470; CG (n = 27): increase to 8.3 at t1, p = 0.47; difference IG—difference CG = − 0.26 (95% CI = − 1.2; 0.6), p = 0.560; Table 2).

#### Depression and anxiety

There was no significant difference regarding the depression subscale (IG (n = 25): 3.7 vs. CG (n = 30): 3.6, p = 0.915; Fig. 1c) or the anxiety subscale (IG (n = 25): 5.4 vs. CG (n = 30): 5.0, p = 0.723; Fig. 1d) between the IG and the CG at t0. Neither depression scores (IG (n = 23): decrease to 3.0 at t1, p = 0.163; CG (n = 28): decrease to 3.3 at t1, p = 1.000; difference IG minus difference CG = − 0.50 (95% CI = − 1.4; 0.4), p = 0.288; Table 2) nor anxiety scores (IG (n = 23): decrease to 4.7 at t1, p = 0.235; CG (n = 28): decrease to 4.6 at t1, p = 0.810; difference IG minus difference CG = − 0.43 (95% CI = − 1.7; 0.8), p = 0.488; Table 2) improved significantly at follow-up in both arms.

#### Willingness-to-pay

The mean one-time willingness to pay for cure did not differ significantly between the IG and the CG at t0 (IG (n = 13): 896€ vs. CG (n = 20): 2268€, p = 0.222; Fig. 1e). The amount of money participants were willing to pay barely changed at follow up (IG (n = 15): increase to 1437€ at t1, p = 0.698; CG (n = 22): decrease to 2264€ at t1, p = 0.066; difference IG minus difference CG = − 234.76 € (95% CI = − 667.9; 198.4), p = 0.277; Table 2).

#### Knowledge quiz

There was no difference in knowledge between the IG and CG at baseline (IG (n = 25): 7.7 vs. CG (n = 30): 7.1, p = 0.209; Fig. 1f). At t1, individual knowledge had not significantly increased for both arms (IG (n = 23): increase to 8.3 at t1, p = 0.164; CG (n = 28): increase to 8.0 at t1, p = 0.423; difference IG minus difference CG = 0.42 (95% CI = 0.3; − 0.4), p = 1.211; Table 2). Mean scores of the individual questions did not differ between the groups at t0. However, at t1 the IG showed a significantly higher score regarding the question on medication that should be avoided during the VIT compared to the CG (IG: 0.36 ± 0.58 vs. CG: 0.11 ± 0.32, p = 0.05; Additional file 3: Table S3).

#### Simulation test

The CG performed significantly better in the simulation test than the IG at t0 (IG (n = 25): 6.7 vs. CG (n = 30): 9.0, p = 0.004; Fig. 1g). At follow up, both arms performed significantly better than at t0 (IG (n = 24): increase to 11.4 at t1, p < 0.0001; CG (n = 28): increase to 10.5 at t1, p = 0.0002). However, the IG showed a significantly higher increase than the CG from t0 to t1 (difference IG minus difference CG = 1.57 (95% CI = 0.4; 2.7), p = 0.008; Table 2).

## Discussion

This prospective single-center pilot study provides data on effects of a structured 90-min educational intervention for patients with insect sting allergy. The intervention included relevant knowledge on insect sting allergy as well as training on practical skills on how to manage anaphylaxis and use the emergency medication correctly. It was performed by allergists in small patient groups in an outpatient setting.

Trained patients and interestingly also the CG had statistically significant benefits with respect to subjective self-assurance in using the emergency medication and patients’ ability to manage an acute sting reaction after study participation. We conclude that outcome improvements may partly be attributable to the repeated testing even in the absence of any education. Furthermore, we cannot rule out that participants of the CG have undertaken self-training as well after study participation at baseline. However, the IG showed a higher gain in self-assurance and ability to manage an acute reaction in a simulation exercise at follow-up, which suggests a possible beneficial effect of the intervention itself regarding these outcomes. Brockow et al. developed a standardized 6-h educational intervention for patients at risk for anaphylaxis. At follow-up, the patient education program was significantly more effective at improving practical emergency management skills compared to no intervention [17]. Gain in self-assurance in using the emergency medication is a very important aspect because uncertainty about the correct use has been reported repeatedly [16, 23]. In a study by Warren et al., not even 60% of patients expressed confidence in their ability to use their EAI correctly [23]. In the mentioned study, 52% of adult participants indicated that no EAI was used during their most severe anaphylactic reaction of which 21% expressed lack of knowledge on when or how to administer epinephrine as leading reason [23]. According to a review on pitfalls in the use of epinephrine for anaphylaxis, inadequate education of patients or providers and uncertainty about when or how to administer epinephrine are frequently reported. However, the most common problem was lack of autoinjector availability [16]. Looking at the willingness to always carry the emergency medication in our study, neither the IG nor the CG showed a significant enhancement at follow-up. As anaphylaxis is potentially life threatening, a prompt availability of medication is essential [16, 24]. A failure or delay in administration of adrenaline may increase the risk of death according to fatality register studies [13]. Nevertheless, noncompliance in carrying the emergency medication is stated repeatedly [2, 9, 10, 14, 16, 17, 23]. However, high rates of willingness to carry the medication were observed at t0 in both of our study arms. Thus, we assume that our patients already paid attention to that necessity before study participation and therefore the effect of the intervention regarding this issue was not significant.

We found no significant improvement regarding depression and anxiety measurements and disease burden (WTP) in both study arms. This observation might reflect the fact that disease related psychological strain was not specifically focused on during our brief 90-min training and psychologists were not involved. However, also Brockow et al., who conducted a longer education program for patients at risk for anaphylaxis with psychologists as part of the team, did not show a significant improvement of anxiety and depression scores of adult participants after the intervention [17]. Furthermore, no significant improvement of disease specific knowledge could be achieved through our 90-min education. In contrast, the more extensive education aforementioned program for the management of anaphylaxis was significantly more effective at improving knowledge compared to no intervention [17]. Improving knowledge seems to be of particular importance. Thus, a systematic review on gaps in anaphylaxis management revealed lack of knowledge as one of the major themes [25]. We suggest that the short intervention period of our study and the monodisciplinary team might be the reason for our negative result. It is conceivable that trained personnel specifically dedicated to effective communication and education (e.g. psychologists, pedagogues) as part of the team might have made a significant difference in this outcome parameter.

There are limitations to our study. The monocentric design and the restricted number of participants limit the generalizability of the results. Due to the limited cohort size, statistical significant differences between subgroups might have been missed or overestimated. However, clinical significance is a decision based on the practical value or relevance of the intervention, and this does not necessarily involve statistical significance as an initial criterion [26]. Additionally, patients with severe reactions (Mueller grade IV) and patients allergic to bee venom were not well represented in the study population. As many beekeepers (a considerable part of the bee allergic population) and patients who experienced more severe reactions are likely to be more aware of the risk and have different habits, the results may not be completely applicable to that subpopulations. Furthermore, we did not investigate long-term effects of the intervention. The lack of randomization is definitely an additional limitation, as patients willing to accept a 90-min training are likely to pay more attention to the management of an acute reaction and probably saw a higher need for improvement, compared to patients not interested in the training. Consistently, the ability to manage an acute sting reaction was significantly better in the CG at t0 than in the IG, while mean values of subjective self-assurance, willingness to always carry the emergency medication, depression and anxiety subscales, willingness to pay and knowledge scores did not differ significantly between the two groups. Moreover, a lack of blinding of the investigator needs to be noted as limitation.

Furthermore, it has to be mentioned, that the recommendations for the components of the “emergency set for self-help “ vary between countries. First line therapy for anaphylactic sting reactions is intramuscular adrenaline, while there is no high-quality evidence to confirm the effectiveness of antihistamines and glucocorticoids in the treatment of anaphylaxis [27]. Thus, in several countries only self-injectable epinephrine is recommended as medical therapy that a patient should carry with them for self-medication of anaphylactic reactions. It is also still a debated issue whether EAI should be carried during and after VIT. In general, patients are protected after reaching the maintenance dose. However, German guidelines recommend carrying an emergency kit after the end of VIT [28].

Overall, our 90-min educational program had a positive impact on the participants’ empowerment to manage an acute sting reaction. It has been shown that patients often have difficulties to recall information provided during brief doctors’ visits [29] and counselling is often neglected [30]. Furthermore, patients seldom express their concerns and often do not ask questions about their diagnosis or treatment during consultations [31, 32]. Educational interventions aim to empower patients in self-management of their disease [33] and an increase in disease knowledge is associated with better therapy adherence and higher patient satisfaction [34, 35]. A recent US study on adults, adolescents and parents of children who had been prescribed an EAI revealed that 61% of participants desired more effective patient education and 47% desired more time dedicated to patient education during the physician visit to improve clinical anaphylaxis management [23]. Beneficial effects of educational programs have been proven for several disorders, e.g. atopic eczema [36], bronchial asthma [37] and anaphylaxis [17].

## Conclusion

We have shown that patients who are willing to invest 90 min in a patient education intervention benefit by an increased subjective and objective empowerment to manage an acute sting reaction.

Based on the data presented here, we believe that brief educational programs for patients with insect sting allergy should be routinely offered to improve patient care.

We think it is not only the responsibility of providers to prescribe emergency medication when indicated but also to train patients with insect sting allergy in its correct use, proper management of a reaction, and to remind patients of the importance of carrying the medication. We recommend a periodical patient education including a simulation training for every patient with insect sting allergy.

## Supplementary Information

**Additional file 1: Table S1.** Knowledge quiz (maximum total score 12 points). Additional details about the "knowledge quiz" are provided.

**Additional file 2: Table S2.** Simulation test (maximum total score 13 points). Additional details about the “Simulation test” are provided.

**Additional file 3: Table S3.** Differences in answering the individual questions of the knowledge quiz between the intervention and the control group at t0 and t1. Differences in answering the individual questions of the knowledge quiz between the IG and CG are provided.

## Data Availability

The datasets used and analysed during the current study are available from the corresponding author on reasonable request.

## Abbreviations

CG: Control group
CI: Confidence interval
EAI: Epinephrine auto-injectors
HADS: Hospital Anxiety and Depression Scale
IgE: Immunoglobulin E
IG: Intervention group
LOCF: Last observation carried forward
max: Maximum
min: Minimum
n: Number
SD: Standard deviation
t0: Baseline
t1: Follow-up
VAS: Visual analogue scales
VIT: Venom immunotherapy
WTP: Willingness-to-pay
