# Supplementary material for: Effects of a 90-min educational intervention for patients with insect venom allergy: a prospective controlled pilot study
Source: Allergy Asthma Clin Immunol. 2021 Feb 25;17:22. doi: 10.1186/s13223-021-00524-7 (PMC7905619; doi:10.1186/s13223-021-00524-7)
Supplement: Supplementary file 1 — Additional file 1: Table S1. Knowledge quiz (maximum total score 12 points). Additional details about the "knowledge quiz" are provided. [file 13223_2021_524_MOESM1_ESM.pdf]

Table S1. Knowledge quiz (maximum total score 12 points)

Question	Response option	Maximum score
**1. Which components are part of your emergency medication set?**	Open-ended question	3 points
**2. What do you have to do, apart from using your emergency medication, in case of an anaphylactic reaction from an insect sting?**	Open-ended question	1 point
**3. Which drugs should be avoided during VIT?**	Open-ended question	2 points
**4. Which active substance does the EAI contain?**	Open-ended question	1 point
**5. Choose the correct answer concerning the storage of the individual substances of the emergency medication set.**	closed multiple-response question	5 points

EAI: Epinephrine auto-injector; VIT: venom immunotherapy.
