# Supplementary material for: Effects of a 90-min educational intervention for patients with insect venom allergy: a prospective controlled pilot study
Source: Allergy Asthma Clin Immunol. 2021 Feb 25;17:22. doi: 10.1186/s13223-021-00524-7 (PMC7905619; doi:10.1186/s13223-021-00524-7)
Supplement: Supplementary file 2 — Additional file 2: Table S2. Simulation test (maximum total score 13 points). Additional details about the “Simulation test” are provided. [file 13223_2021_524_MOESM2_ESM.pdf]

Table S2. Simulation test (maximum total score 13 points)

**Hypothetical scenario:**	
You are alone in the forest. You notice wheals all over your body, feel dizzy and you are short of breath minutes after an insect sting. How do you react?	
**Evaluation criteria**	**Score**
**1. Look for/unpack the emergency medication set**	Immediately: 1 point
**2. Use of all three components of the emergency medication set in the correct order**	range: 0 (incomplete use) to 3 (use of all components, EAI at first)
**3. Proper administration of an EAI training device**	range: 0 (wrong administration) to 3 (correct administration)
**4. Seek help**	range: 0 (not seeking help) to 3 (seeking emergency medical attention)
**5. Time until completion of the test**	range: 0 (> 120 seconds) to 3 (< 30 seconds)

EAI: Epinephrine auto-injector.
