# Supplementary material for: Effects of a 90-min educational intervention for patients with insect venom allergy: a prospective controlled pilot study
Source: Allergy Asthma Clin Immunol. 2021 Feb 25;17:22. doi: 10.1186/s13223-021-00524-7 (PMC7905619; doi:10.1186/s13223-021-00524-7)
Supplement: Supplementary file 3 — Additional file 3: Table S3. Differences in answering the individual questions of the knowledge quiz between the intervention and the control group at t0 and t1. Differences in answering the individual questions of the knowledge quiz between the IG and CG are provided. [file 13223_2021_524_MOESM3_ESM.pdf]

Table S3. Differences in answering the individual questions of the knowledge quiz between the intervention and the control group at t0 and t1

Question	Intervention Group	Control Group	p
**1. Which components are part of your emergency medication set? (max. 3 points)**			
t0 (mean ± SD)	1.96 ± 1.21	1.77 ± 1.2	0.55[a]
t1 (mean ± SD)	2.32 ± 1.21	1.89 ± 1.07	0.19[a]
**2. What do you have to do, apart from using your emergency medication, in case of an anaphylactic reaction from an insect sting? (max. 1 point)**			
t0 (mean ± SD)	0.88 ± 0.33	0.73 ± 0.45	0.18[a]
t1 (mean ± SD)	0.95 ± 0.21	0.93 ± 0.26	0.71[a]
**3. Which drugs should be avoided during VIT? (max. 2 points)**			
t0 (mean ± SD)	0.20 ± 0.41	0.10 ± 0.40	0.37[a]
t1 (mean ± SD)	0.36 ± 0.58	0.11 ± 0.32	**0.05**[a]
**4. Which active substance does the EAI contain? (max. 1 point)**			
t0 (mean ± SD)	0.64 ± 0.49	0.57 ± 0.50	0.59[a]
t1 (mean ± SD)	0.86 ± 0.35	0.68 ± 0.48	0.13[a]
**5. Choose the correct answer concerning the storage of the individual substances of the emergency medication set. (max. 5 points)**			
t0 (mean ± SD)	3.92 ± 0.91	3.90 ± 0.96	1.00[b]
t1 (mean ± SD)	3.73 ± 0.83	3.86 ± 1.11	0.88[b]

Differences were tested for significance with [a] unpaired t-test or [b] paired t-test. EAI: Epinephrine auto-injector; max.: maximum; SD: standard deviation; t0: baseline; t1: follow-up. Significant findings are highlighted in bold.
